# Editorial: T cell metabolism in infection

**DOI:** 10.3389/fimmu.2022.956876

**Published:** 2022-08-03

**Authors:** Jin-Fu Xu, Ying-Zhou Xie, Duo-Jiao Wu

**Affiliations:** ^1^ Department of Respiratory and Critical Care Medicine, Shanghai Pulmonary Hospital, School of Medicine, Tongji University, Shanghai, China; ^2^ Institute of Respiratory Medicine, School of Medicine, Tongji University, Shanghai, China; ^3^ Zhongshan Hospital, Fudan University, Shanghai, China

**Keywords:** T cell, infection, metabolism, therapeutics, Tfh

T cells play a central role in the adaptative immunity to counter both bacterial and viral infections. They are derived from hematopoietic stem cells in bone marrow and develop in the thymus. Mature T cells can be classified into CD4+ T helper cells and CD8+ cytotoxic T cells, according to the surface marker and physiological function. The T cell differentiation is a comprehensive outcome of transcriptional, metabolic, and epigenetic changes. Following the pathogen clearance, most of the effector T cells are programed to die by means of apoptosis (1). The small proportion that remains develops into memory T cells, which can activate rapidly and strengthen to execute their effector function in response to the superinfection. T cells at each differentiation stage call for a different energy supply to match their specific function. The two major metabolic pathways, oxidative phosphorylation (OXPHOS) and glycolysis, generate energy at distinct rates and efficiency. Thus, the metabolic reprogramming is critical in the T cell proliferation, clonal expansion, and differentiation (2). Metabolism is not only a critical process in order to provide energy but can also be an approach for cells to get the chemical groups for transcriptional and post-translational modification (3, 4). The T cell exhaustion caused by metabolic dysfunction may result in the intractable chronic infection, highlighting the importance of metabolic homeostasis in the efficient control of infection (5, 6). This Research Topic mainly discusses advanced research on the metabolic mechanisms of the T cells’ ability to fight infections. The potential approaches and applications in which T cells might be manipulated by reprogramming the metabolic pathways for therapeutic purposes are also discussed.

Wik and Skalhegg provided a systemic review of the T cell function and the molecular basis of the T cell activation. The differentiated T cells can work together with other immune cells, for example the macrophages and B cells, to eliminate infections. Generally, naïve CD8+ T and CD4+ Th0 cells, in a quiescent state, mainly employ OXPHOS to maintain the homeostasis. Once activated *via* TCR ligation, the T cells convert the metabolic pathway into glycolysis, where ATP production is nearly 100 times faster than OXPHOS, as well as glutaminolysis and fatty acid oxidation (FAO) to support the effector function. The mechanistic target of rapamycin complexes (mTORC), mTORC1, and mTORC2 are key players to connect the antigen-activated signaling pathway with the metabolic reprogramming. The disrupted ligation can break the T cell differentiation balance and subsequently, the immune disorder. The original research conducted by Wei et al. reported that the knockout of the G protein-coupled receptor GPR174 can reduce gut inflammation and impact the CD11c+ dendritic cells. The knockout of GPR174 in LPS-primed BMDCs expressed less MHC-II, which may affect the T cell metabolism *via* ligation and result in less CD4+ naïve T cell proliferation and differentiation to effector Th1 cells.

The metabolism alteration caused by SARS-CoV-2 and *Mycobacterium tuberculosis* (*Mtb*) infection were reviewed by Wik and Skalhegg. SARS-CoV-2 uses its ligation to receptor angiotensin-converting enzyme 2 (ACE2) to induce the metabolic reprogramming of host cells. The ligation increased the glycolytic rate in both peripheral blood mononuclear cells (PBMCs) and monocytes to promote its intracellular replication but was found inhibitory to the glycolytic activity of effector T cells. Evidence from different studies seemed contradictory, as Wik and Skalhegg concluded that T cell metabolism was differentially affected in the mild phase compared to moderate and severe disease. *Mtb* infections contribute to decreased T cell glycolytic metabolism through several mechanisms, for example the increased expression of both PD-1 and CTLA4 on the T cell surface. The high-level expression of these inhibitory receptors correlated with decreased glucose uptake, reduced glycolysis, and mitochondrial respiration. Both early and long-term *Mtb* infection can induce distinct defects to the metabolism of CD8+ T cells. Mitochondrial cyclophilin D (CypD) was identified as an important checkpoint in T cell immunity against *Mtb* infection by regulating mitochondrial ROS to promote T cell proliferation and function. The loss of CypD can induce metabolic changes, showing an increase in both OXPHOS and glycolysis, and resulting in the extreme susceptibility to *Mtb* infection. This highlighted the critical role of T cell metabolism in the tolerance to *Mtb* infection.

The continuous stimulation by pathogens can lead T cells to enter a stage of metabolic dysfunction where oxidative metabolism is dampened and finally cause T cell exhaustion. Zheng et al. provided a review focused on how the metabolic dysfunction led to T cell exhaustion in the chronic viral infection. The persistent stimulation by the virus increased the expression of inhibitory receptors like PD-1 and CTLA-4. The ligation to inhibitory receptors subsequently attenuated the PI3K/AKT/mTORC signaling, resulting in the decrease of glucose uptake and glycolysis. The PD-1 ligation also promoted the expressions of multiple enzymes involved in FAO and mitochondrial respiration to reprogram fatty acid metabolism. Additionally, the PD-1 ligation altered the amino acid metabolism of effector T cells. All the dysfunction of metabolisms can lead to the T cell exhaustion and chronic infection. Based on the finding of shifted cholesterol levels in HCV infections and the role of SREBP in HIV within-CD4+ T cell replication, Zheng et al. proposed the hypothesis that cholesterol metabolism may be crucial in T cell exhaustion during chronic viral infection, however, further evidence is demanded.

T follicular helper (Tfh) cells support the B cells in the germinal center to secret antimicrobial antibodies, thus it is an important subset for T cells to fight against infections. Mayberry et al. provided a comprehensive review of the Tfh cells’ differentiation and the metabolism reprogramming in this process, especially the role of Bcl-6 and ICOS in balancing the expression of glycolysis and OXPHOS related genes. The signaling mediated by mTORC1 and mTORC2 is a key link to the ICOS regulatory pathway (7). However, the infection-induced metabolic reprogram specific to Tfh cells is not sufficiently elucidated and needs to be extensively explored.

The micronutrient is important in the maintenance of the cell metabolism and thymus microenvironment. Sergi reviewed some clinical trials to investigate the benefit of zinc in patients receiving hematopoietic stem cell transplantation (HSCT) in individuals affected with other immunologic deficiencies. Zinc has been identified as crucial to the maintenance of the physiological function of thymus and the entire immune system. It can also act on transcription factors such as the NF-κB and AP-1, which are predominant in the infection-related inflammation signaling pathways. However, as the mechanistic data are still lacking, he posted the opinion that the role of zinc in the infection to reshape the T cell metabolic process deserved further investigation as it is among the most abundant trace minerals in the human body.

Overall, the articles included in this Research Topic comprehensively summarized the critical roles of metabolic homeostasis and reprogramming in T cell proliferation and differentiation. The key molecules and signaling pathway participating in T cell metabolism were also detailed. We hope the discussion under this topic will inspire the development of novel T cell metabolism-targeted therapeutic approaches.

## Author contributions

J-FX contributed to the concern and design of this editorial. J-FX and Y-ZX contributed to the writing of it. D-JW revised it. All authors contributed to the article and approved the submitted version.

## Funding

National Natural Science Fund for Distinguished Young Scholar (No. 81925001) and Key Scientific Innovation Project of Shanghai Municipal Education Commission (No. 202101070007-E00097).

## Conflict of interest

The authors declare that the research was conducted in the absence of any commercial or financial relationships that could be construed as a potential conflict of interest.

## Publisher’s note

All claims expressed in this article are solely those of the authors and do not necessarily represent those of their affiliated organizations, or those of the publisher, the editors and the reviewers. Any product that may be evaluated in this article, or claim that may be made by its manufacturer, is not guaranteed or endorsed by the publisher.
